# Footwear choices and their association with plantar fasciitis among adult women in Saudi Arabia: a cross-sectional study

**DOI:** 10.1038/s41598-025-24122-4

**Published:** 2025-11-17

**Authors:** Reem M. Alwhaibi, Gopal Nambi, Farah Alharbi, Huda bin Obaid, Layan Alnasser, Manal Akram, Layan Al Qhatani

**Affiliations:** 1 Department of Rehabilitation Sciences, College of Health and Rehabilitation Sciences, Princess Nourah Bint Abdulrahman University, P.O. Box 84428, 11671 Riyadh, Saudi Arabia; 2 Department of Health and Rehabilitation Sciences, College of Applied Medical Sciences, Prince Sattam Bin Abdulaziz University, Al-Kharj, Saudi Arabia

**Keywords:** Plantar fasciitis, Footwear choices, Shoe types, Footwear habits, Foot pain, Heel pain, Plantar pain, Adult women, Risk factors, Signs and symptoms

## Abstract

Plantar fasciitis (PF) is a common foot condition in adult women, influenced by footwear choices. Although risk factors for PF have been studied, limited research has focused on the link between footwear choices and PF symptoms among Saudi women. Cultural habits, such as frequent use of high heels and sandals, may contribute to PF, highlighting the importance of understanding this relationship for effective prevention and management. This study aimed to examine the influence of footwear choices on the prevalence of PF among adult women in Saudi Arabia to inform awareness campaigns and guide appropriate footwear recommendations. This cross-sectional study examined the impact of footwear choices on PF symptoms among 401 adult women in Saudi Arabia. Data were collected over period of 8 weeks using an online questionnaire incorporating the Foot Health Status Questionnaire (FHSQ). Descriptive and inferential statistical analyses were conducted using SPSS to assess associations between footwear types and PF symptoms. PF-related symptoms were highly prevalent, with 58.4% of participants reporting pain after prolonged standing, no significant associations were found between general shoe type and PF symptoms (*p* > 0.05). However, significant associations emerged between pain after standing and the frequency of high heel use (*p* = 0.047), as well as between shoe type and the regular use of high heels or thick soles (*p* = 0.046). PF was also linked to significantly greater functional limitations and lower quality of life scores (Cohen’s d =  − 2.40). Frequent use of high heels was significantly linked to pain after standing, a key symptom of PF, while general shoe type showed no direct association. This emphasizes the importance of assessing specific footwear habits rather than broad categories when evaluating PF risk. Encouraging the use of supportive footwear with proper arch support and cushioning may reduce PF symptoms. Future studies should focus on culturally appropriate footwear interventions.

**Supplementary Information:**

The online version contains supplementary material available at 10.1038/s41598-025-24122-4.

## Introduction

Plantar fasciitis (PF) is a prevalent musculoskeletal disorder characterized by inflammation of the plantar fascia, a thick band of connective tissue that supports the foot arch. The condition is most commonly associated with severe heel pain, particularly during the first steps in the morning or after prolonged standing or walking. This discomfort results from repetitive microtrauma at the fascia’s insertion site, leading to a degenerative rather than purely inflammatory process^1^ PF accounts for approximately 11–15% of all foot complaints requiring professional care and is a leading cause of heel pain in both athletic and non-athletic populations^2^. Adults, particularly women, appear to be disproportionately affected, possibly due to a combination of biomechanical, hormonal, and lifestyle factors^3^.

Although clinical and biomechanical aspects of PF have been well-documented in the literature, emerging evidence emphasizes the role of extrinsic factors, especially footwear choices, in the onset and exacerbation of PF symptoms. Poorly constructed shoes lacking arch support, cushioning, and proper fit can contribute to increased heel loading and plantar fascia strain. Umar et al., for example, reported that 83.2% of patients diagnosed with PF in Pakistan wore inappropriate footwear, typically thin-soled, flat shoes without heel elevation or arch support, while only 16.8% wore shoes with design features known to mitigate heel pain^4^. Similarly, Belhan et al. found that thinning of the heel fat pad, aggravated by unsuitable footwear, was strongly associated with plantar heel discomfort^5^. This evidence demonstrates that footwear is not merely a passive factor but an active contributor to plantar fascia strain and pain development, especially in populations with prolonged weight-bearing demands.

Beyond pain severity, footwear has been linked to broader functional and psychosocial outcomes. Sullivan et al. demonstrated that individuals with plantar heel pain, especially women, report significantly more difficulties with footwear comfort, fit, and selection compared to healthy controls^6^. These issues are often associated with reduced plantar loading, impaired toe flexor strength, and altered gait dynamics. Furthermore, suboptimal footwear has been shown to hinder daily mobility and reduce quality of life (QoL) scores across multiple domains.

In Saudi Arabia, PF is increasingly recognized as a public health concern. Khired et al. reported a prevalence of 37% in the general population, with middle-aged adults (40–55 years) experiencing significantly higher risk^7^. These region-specific findings suggest a need to examine unique environmental and cultural contributors to PF. Notably, few studies have examined the impact of cultural and lifestyle determinants, such as preferred footwear types, on PF among Saudi women, despite the country’s unique climatic, dress, and social norms. In daily life, many Saudi women wear sandals, flat shoes, or high heels for extended periods. These types of shoes are often characterized by inadequate heel support, lack of arch contouring, or excessive heel elevation, potentially exacerbating PF symptoms. These region-specific lifestyle habits, combined with limited local research, underscore the need for culturally relevant investigations.

Region-specific research further highlights the influence of socio-economic and demographic factors on PF outcomes. Alshammari et al., using the Foot Health Status Questionnaire (FHSQ), evaluated 209 PF patients in Saudi Arabia and found that older adults, women, unemployed individuals, and those with low income experienced significantly worse outcomes in foot pain and footwear-related domains^8,9^. The findings underscore the need for tailored footwear interventions to improve the quality of life, particularly among disadvantaged subgroups. This evidence reinforces the multidimensional role of footwear in both the clinical presentation and social burden of PF. Unlike Alshammari et al., which assessed HRQoL impairment in both male and female PF patients, our study specifically examined how footwear choices influence PF prevalence in adult women in Saudi Arabia. Also, using binary logistic regression, we identified footwear-related predictors, providing novel evidence to guide awareness campaigns and footwear recommendations.

While previous literature highlights that middle-aged adults (40–55 years) are at a higher risk for PF, we broadened our target population to include all adult women (≥ 18 years) to capture a wider spectrum of footwear habits and their potential association with PF. This decision was based on two considerations: (1) PF has also been reported in younger and older adults at varying prevalence rates and (2) our aim was to provide findings that could inform preventive strategies and awareness campaigns relevant to the entire adult female population in Saudi Arabia, not only those in the middle-aged subgroup.

The novelty of this study lies in its culturally contextualized investigation of the association between footwear choices and PF symptoms among adult women in Saudi Arabia. While prior research has broadly associated footwear with PF development, no study to date has examined the specific impact of commonly worn shoe types in this demographic, nor has it explored how local preferences, shaped by fashion, climate, and religious attire, may interact with biomechanical risk factors.

Therefore, the present study aims to (1) identify the types of shoes most commonly worn by adult Saudi women, (2) assess the prevalence of PF symptoms in this population, and (3) determine whether specific shoe types are associated with increased PF risk. The findings may contribute to better clinical decision-making, culturally appropriate public health messaging, and footwear industry design guidance tailored for women in the Middle East. By addressing a significant gap in region-specific literature, this research has the potential to inform targeted educational interventions, guide footwear recommendations, and ultimately improve pain management and quality of life for affected individuals.

## Methodology

### Study design

A cross-sectional design was used to examine the impact of footwear choices on PF symptoms in adult women in Saudi Arabia. This design was selected to assess the prevalence of variables and their associations at a single point in time. The study was conducted over a period of 8 weeks. Ethical approval was obtained from the Institutional Review Board (IRB) at Princess Nourah University (PNU-IRB), Approval number: 25-0001.

### Participants

#### Inclusion/exclusion criteria

All participants included were women aged 20 and older, living in Saudi Arabia. inclusion criteria: (1) Self-reported symptoms consistent with PF^10^, including morning pain that was most intense with the first few steps after waking up, stabbing pain at the bottom of the heel on the inner side, pain after long periods of standing or sitting, and heel swelling or tenderness . (2) Regular use of footwear such as well-cushioned shoes or high heels. (3) Asymptomatic participants were included as control if they reported no past or present history of plantar heel pain or related symptoms. Exclusion criteria: (1) Participants who had undergone foot surgery; (2) those diagnosed with musculoskeletal disorders such as systemic arthritis, neurological conditions, lumbar radiculopathy, or diabetes-related neurological or vascular foot complications, including peripheral neuropathies.

#### Sample size calculation

According to the General authority for statistics (2022), the total population of adult women in SA is 5,234,177 Using OpenEpi software (version 3.01), and assuming a 95% confidence interval with a 5% margin of error, the required minimum sample size was calculated to be 385 participants. To account for potential exclusions and incomplete responses, we aimed to recruit approximately 400 participants in total^11^.

#### Sampling method

The sampling method used in this study was convenience sampling. Participants were recruited through online distribution channels over a duration of 8 weeks. This approach allowed for rapid and cost-effective data collection from adult women in Saudi Arabia who were accessible and willing to participate. While this method does not ensure equal probability of selection or full population representativeness, it is commonly employed in exploratory public health research and provided sufficient data to analyze associations between footwear choices and PF symptoms.

#### Data collection tools

The Arabic version of Foot Health Status Questionnaire (FHSQ-Ar) was selected as the primary data collection tool for this study due to its comprehensive and validated design, which aligns with the research objectives.

The FHSQ is a self-administered questionnaire that assesses multiple dimensions of foot health, including foot pain, foot function, footwear satisfaction, and general foot health, with each domain scored on a scale from 0 to 100. Higher scores indicate better outcomes, allowing for a detailed evaluation of specific aspects of foot health. The Footwear ease of finding well-fitting shoes, and any restrictions in the variety of footwear that can be worn. The scores generated for this domain range from 0 to 100, with lower scores indicating significant difficulties with footwear comfort, fit, and selection, and higher scores suggesting no challenges in obtaining appropriate footwear^8^.

The FHSQ-Ar contains 13 core items grouped into four domains: foot pain (4 items), foot function (4 items), footwear (3 items), and general foot health (2 items). All items use ordinal Likert-type scales with 5 response options, except for two general health items, which use categorical responses (e.g., “excellent”, “good”, “fair”, “poor”). Domain scores were computed according to the FHSQ manual and treated as continuous variables (0–100 scale) for statistical analyses.Within the foot function domain, one item specifically assesses the extent to which foot health limits stair use. This item is part of the validated FHSQ instrument and was analyzed as a continuous score (0–100), with higher scores indicating fewer functional limitations.

Additional items were included to collect data on footwear habits and demographic characteristics. Footwear variables included daily shoe type (categorical), frequency of high-heel use (ordinal: daily, weekly, occasionally, never), average heel height (ordinal: < 2 cm, 2–5 cm, > 5 cm), and hours of wear per day (ordinal: < 2 h, 2–4 h, 5–7 h, > 7 h). Demographic variables included age, height, and weight (continuous), and occupation (categorical). These classifications confirm the ordered nature of the relevant footwear variables and support their use in trend analyses.The FHSQ is a validated tool with adequate internal consistency, ranging from 0.70 to 0.92, and reliability values between 0.69 and 0.80, ensuring the accuracy and consistency of the data collected. Its structure has been widely validated for use in diverse populations and foot-related conditions, making it suitable for assessing the relationship between footwear choices and PF in this demographic. The questionnaire’s adaptability also allows for the inclusion of culturally specific examples, ensuring relevance to the Saudi context, particularly the frequent use of sandals and high heels.

#### Procedure

The data collection for this study was conducted using an online questionnaire developed through Google Forms, ensuring a user-friendly interface for participants. The questionnaire was divided into three main sections: introduction and consent, eligibility screening, and the validated tool. The first screen provided participants with a detailed introduction to the study, including its purpose, objectives, and the importance of their contributions. Participants were informed that their participation is voluntary, their responses would remain anonymous and confidential, and they could withdraw at any time. A mandatory consent question followed, requiring participants to indicate their agreement to proceed. Those who did not consent were redirected to a thank-you message and exited the form.

Eligible participants were identified through a series of yes/no questions that assessed compliance with the inclusion criteria (e.g., being aged 20 years and older, and self-reporting symptoms consistent with PF such as morning heel pain or pain after standing ) and excluded those with confounding conditions such as foot surgeries or systemic arthritis. Participants were not clinically examined; instead, PF status was determined through self-reported symptoms using a checklist embedded in the screening section. Participants who met the eligibility criteria were then directed to the FHSQ. The footwear-specific section of the FHSQ was customized to reflect culturally relevant footwear preferences, such as sandals and high heels, commonly worn by Saudi women.

Participants were recruited through various online platforms, including social media channels and professional networks, using a study description and a link to the Google Form to facilitate accessibility and convenience. Upon completing the questionnaire, participants submitted their responses, which were automatically recorded and securely stored in the Google Forms database. Real-time monitoring of responses was conducted to ensure data completeness and quality. Incomplete submissions or those from ineligible participants were excluded during the data cleaning process.

#### Data analysis

Data were analyzed using IBM SPSS Statistics (Version 30.0; IBM Corp., Armonk, NY, USA). Both descriptive and inferential statistical methods were used to address the study objectives. Descriptive statistics were applied to summarize participants’ demographic data, footwear patterns, and the prevalence of PF-related symptoms. Continuous variables were presented using means, standard deviations, medians, and interquartile ranges. Categorical variables were reported as frequencies and percentages.

Chi-square tests were used to assess associations between categorical variables, such as footwear type and the presence of PF symptoms. Where expected frequencies in contingency tables were low, the likelihood ratio chi-square was reported as an alternative to Pearson’s chi-square, providing a more reliable estimate under these conditions. For variables with a natural order (e.g., frequency of high-heel use, heel height categories, hours worn per day), the linear-by-linear association test (Mantel–Haenszel test for trend) was applied to evaluate monotonic trends across ordered categories, which is appropriate when at least one variable is ordinal.

Binary variables (e.g., presence or absence of PF-related symptoms) were treated as dichotomous ordinal variables (0 = No, 1 = Yes) when applying the linear-by-linear association test. This approach is statistically appropriate because binary variables can be conceptualized as ordinal with two ordered categories, allowing for the detection of monotonic trends when analyzed against an ordinal predictor.

Independent-samples t-tests were performed to compare functional limitations and quality of life scores between participants with and without PF. Binary logistic regression was used to examine whether footwear-related factors predicted the presence of PF, adjusting for body mass index (BMI, continuous, kg/m^2^) and physical activity level (categorical: low, moderate, high) as covariates . Statistical significance was determined at *p* < 0.05 with a 95% confidence interval.

In the binary logistic regression, “type of footwear” was entered as a single categorical predictor using dummy variables with sports shoes as the reference category (Ref). The remaining levels (high heels, flat shoes, sandals, traditional shoes) were compared to this reference. Ordered exposure variables were modeled as ordinal scores to capture monotonic dose–response: average heel height (1 =  < 2 cm, 2 = 2–5 cm, 3 =  > 5 cm), frequency of high-heel use (0 = never, 1 = occasionally, 2 = weekly, 3 = daily), and hours wearing the primary shoes per day (1 =  < 2 h, 2 = 2–4 h, 3 = 5–7 h, 4 =  > 7 h). Use of cushioned insoles or orthotics was coded as binary (0 = no, 1 = yes). This parameterization yields one coefficient per ordered predictor (trend across increasing categories) and one coefficient per footwear type relative to the reference.

## Results

**Fig. 1:**
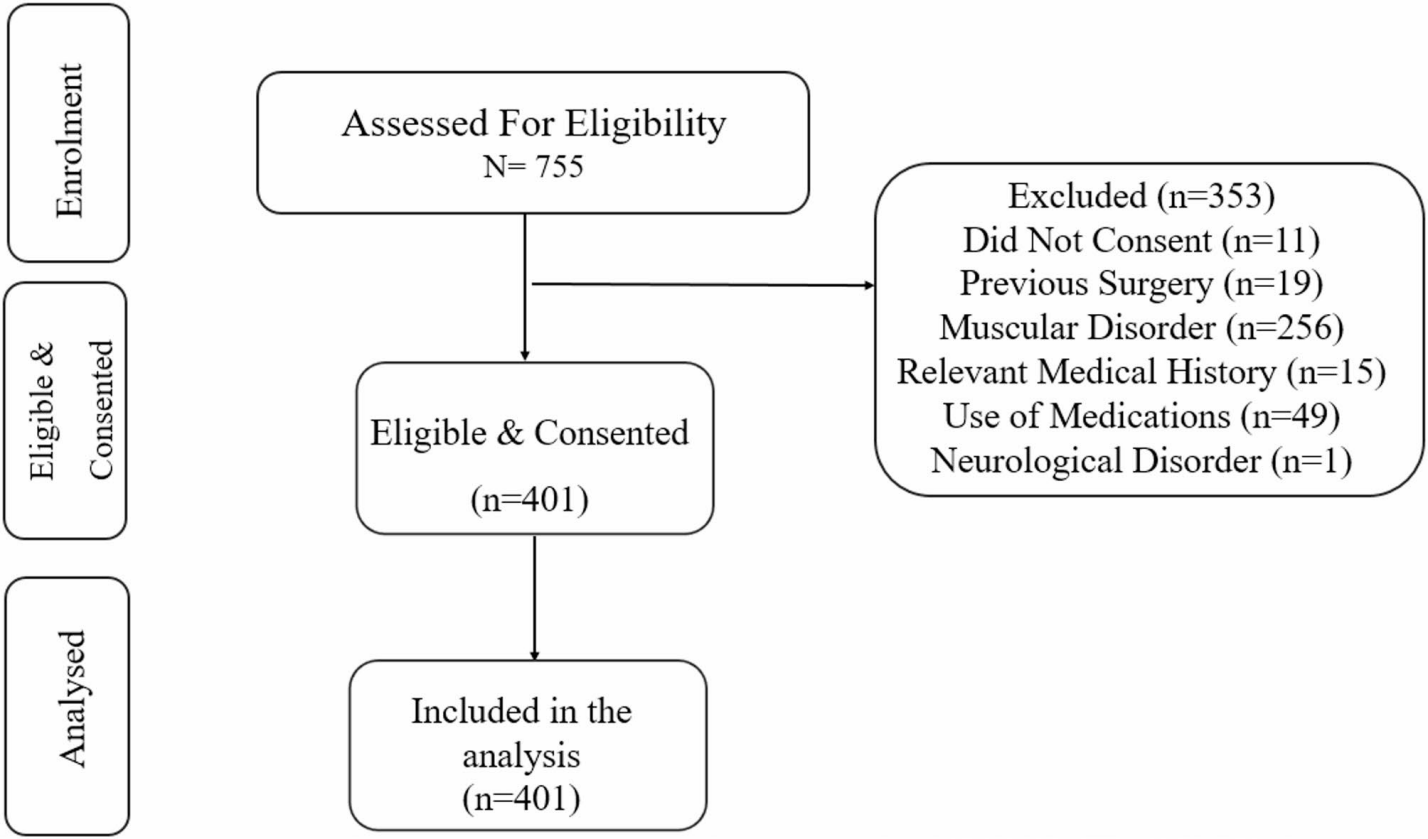
Flow diagram for eligibility assessment and enrolment.

A total of 755 women initially responded to the recruitment call and accessed the online questionnaire. Of these, 353 were excluded based on predefined eligibility criteria, including lack of consent, history of foot surgery, musculoskeletal or neurological disorders, relevant medical conditions, or medication use. Consequently, 401 participants met the inclusion criteria and were retained for the final analysis. The detailed screening and enrollment process is illustrated in Fig. 1.

As presented in Table 1, the majority of participants (82%) fell within the 20–35 age group. The mean age, height and weight of participants were 28 years, 157.73 ± 15.30 cm and 65.89 ± 17.23 kg, respectively. Students represented the largest occupational group (65.6%). Sports shoes were reported as the most commonly worn type on a daily basis (75.3%), followed by traditional and flat shoes. Participants who selected “Other” described a variety of specialized footwear, including medical grade orthopedic shoes with short heels either flat or with a forefoot lift, clog shoes, safety shoes commonly used in industrial work environments, and boots prescribed by specific medical brands for foot support. In addition, some participants reported alternating between house slippers indoors and athletic shoes outdoors, depending on the setting.

**Table 1:** Demographic characteristics and footwear habits of adult female participants (n = 401).

Variable	Category	Frequency (n)	Percentage (%)	Mean ± SD
Age	20–35	329	82	28 ± 12.16
	36–50	65	16	42 ± 9.67
	51–60	8	1.99	28.5 ± 15.74
Profession	Employed	86	21.40	
	Housewife	27	6.70	
	Student	263	65.60	
	Unemployed	25	6.20	
Shoe type	High heel	11	2.70	
	Flat shoes	33	8.20	
	Sports shoes	302	75.30	
	Sandals	7	1.70	
	Traditional	37	9.20	
	Other	11	2.70	
Height (cm)				157.73 ± 15.30
Weight(kg)				65.89 ± 17.23

Table 2 shows the prevalence of self-reported PF-related symptoms , among participants, 58.4% reported pain in the soles of the feet after standing for prolonged periods. Severe morning pain was reported by 16.0%, stabbing heel pain by 15.7%, and heel swelling or sensitivity by 8.2%.

**Table 2:** Prevalence of self-reported plantar fasciitis symptoms among adult female participants (n = 401).

Symptom	Response	Frequency (n)	Percentage (%)
Heel swelling or sensitivity	No	368	91.8
	Yes	33	8.2
Stabbing heel pain	No	338	84.3
	Yes	63	15.7
Sole pain after standing	No	167	41.6
	Yes	234	58.4
Severe morning pain	No	337	84
	Yes	64	16

To explore footwear-related factors from two behavioral angles, we conducted two chi-square analyses. The first (Table 3) assessed the association between participants’ primary daily shoe type and PF symptoms. The results indicated no statistically significant relationships between shoe type and stabbing heel pain, χ^2^ (5, N = 401) = 3.67, *p* = 0.598; heel swelling or sensitivity, χ^2^ (5, N = 401) = 1.99, *p* = 0.851; or pain after standing for long periods, χ^2^ (5, N = 401) = 3.30, *p* = 0.653. However, there was a statistically significant association between shoe type and the regular use of thick-soled or high-heeled shoes, χ^2^ (5, N = 401) = 11.27, *p* = 0.046.

**Table 3:** Chi square analysis of the association between general daily shoe type and footwar-related behaviors and plantar fasciitis-related symptoms (n = 401).

Symptom	Chi-square (χ^2^)	*p*-value	Likelihood ratio	LR *p*-value	Linear-by-linear association	Linear Assoc. *p*-value
Footwear behavior						
High heel use	11.268	0.046	12.56	0.028	0.048	0.827
Plantar fasciitis symptoms						
Pain after standing	3.303	0.653	3.254	0.661	0.002	0.961
Heel swelling or sensitivity	1.985	0.851	3.434	0.633	0.141	0.707
Stabbing heel pain	3.667	0.598	3.346	0.647	2.213	0.137
Sever morning pain	2.412	0.661	2.295	0.681	0.328	0.567

Likelihood Ratio: alternative chi-square statistic used when expected frequencies are small.

Linear-by-Linear Association: chi-square test for trend across ordinal categories.

“High heel use” represents a footwear behaviour, not a symptom. Other variables listed correspond to self-reported PF-related symptoms.

The second (Table 4) focused on the frequency of high heel use, regardless of whether heels were the primary footwear. The results indicated a significant association was found between the frequency of high heel use and pain after standing, χ^2^ (3, N = 401) = 7.94, *p* = 0.047. In contrast, the frequency of wearing high heels was not significantly associated with stabbing heel pain, χ^2^ (3, N = 401) = 0.99, *p* = 0.804; heel swelling or sensitivity, χ^2^ (3, N = 401) = 1.58, *p* = 0.663; or severe morning pain, χ^2^ (3, N = 401) = 2.55, *p* = 0.466 (Table 4).

**Table 4:** Chi-square analysis of frequency of high heel use and plantar fasciitis symptoms (n = 401).

Symptom	Chi-square (χ^2^)	*p*-value	Likelihood ratio	LR *p*-value	Linear-by-linear association	Linear Assoc. *p*-value
Pain after standing long periods	7.943	0.047	7.847	0.049	0.465	0.495
Severe morning pain on first steps	2.552	0.466	2.377	0.498	0.664	0.415
Stabbing pain inside heel	0.989	0.804	1.054	0.788	0.019	0.891
Swelling or sensitivity in heel area	1.584	0.663	1.373	0.712	0.482	0.487

Likelihood Ratio: alternative chi-square statistic used when expected frequencies are small.

Linear-by-Linear Association: chi-square test for trend across ordinal categories.

A binary logistic regression was performed to determine whether footwear-related factors could predict the presence of PF. The dependent variable was self-reported PF symptoms (1 = PF present, 0 = PF absent). Predictor variables included daily shoe type, average heel height, frequency of high heel use, hours worn per day, and use of cushioned insoles or orthotics . In the regression model, “type of footwear” was dummy-coded with sports shoes as the reference category; ordered exposure variables (heel height, frequency of high-heel use, and hours worn per day) were entered as ordinal scores to evaluate linear trend. The model was adjusted for body mass index (BMI, continuous) and physical activity level (categorical: low, moderate, high), both of which were included as covariates.

The overall regression model was not statistically significant, χ^2^ (9, N = 401) = 8.35, *p* = 0.500, indicating that the combined predictors did not significantly differentiate between individuals with and without PF. Neither BMI nor physical activity level were significantly associated with PF symptoms (*p* = 0.243 and *p* = 0.489/0.262 for moderate and high activity, respectively). Similarly, none of the footwear-related predictors reached statistical significance at the 0.05 level. Although some predictors, such as use of cushioned insoles or orthotics (*p* = 0.074), showed odd ratios greater than 1.0, these findings should be interpreted with caution as they lack statistical support. These results suggest that, within this sample, commonly reported footwear characteristics and usage patterns were not significant predictors of PF. The full regression results are presented in Table 5.

**Table 5:** Binary logistic regression analysis predicting plantar fasciitis symptoms based on footwear type and usage patterns (n = 401).

Variable	β	SE (β)	Wald’s χ^2^	*p*-value	Odds ratio (e)
Type of footwear* (Ref: sports shoes)					
High heels	0.127	0.935	0.018	0.892	1.135
Flat shoes	0.174	0.846	0.042	0.837	1.19
Sandals	0.622	0.901	0.477	0.49	1.862
Traditional shoes	− 0.062	1.143	0.003	0.957	0.940
Footwear characteristics/behaviors					
Average heel height	0.237	0.219	1.178	0.278	1.268
Frequency of high heel use	0.073	0.242	0.09	0.764	1.076
Hours wearing basic shoes	− 0.181	0.146	1.533	0.216	0.834
Use of cushioned insoles or orthotics	0.592	0.331	3.19	0.074	1.807
Body mass index (kg/m^2^)	0.021	0.018	1.361	0.243	1.021
Physical activity level (Ref: low)					
Moderate	− 0.287	0.415	0.478	0.489	0.750
High	− 0.531	0.473	1.258	0.262	0.588
Constant	− 1.673	1.171	2.041	0.153	0.187

An independent-samples t-test was conducted to compare the extent to which foot health limits stair use between those with and without PF. Results indicated a statistically significant difference, t(399) = 7.18, *p* < 0.001, with individuals with PF (M = 1.30 ± 1.13) reporting significantly greater limitations than those without PF (M = 0.53 ± 0.89). Although normality tests (Shapiro–Wilk and Kolmogorov–Smirnov) indicated deviations from normality in both groups (*p* < 0.001), the large sample size supports the robustness of the t-test results . Therefore, the t-test findings remain valid despite non-normal distributions.

Functional limitations were compared between participants with and without PF using an independent-samples t-test. As shown in Table 6, individuals with PF reported significantly greater limitations in stair use compared with those without PF, t(399) = 7.18, *p* < 0.001 (PF: M = 1.30 ± 1.13; non-PF: M = 0.53 ± 0.89). The effect size for the difference in functional limitations was medium-to-large (Cohen’s d = 0.78), indicating a meaningful functional impact among participants with PF. Although normality tests (Shapiro–Wilk and Kolmogorov–Smirnov) indicated deviations from normality in both groups (*p* < 0.001), the large sample size supports the robustness of the t-test results, and findings remain valid despite non-normal distributions.

**Table 6:** Independent-samples t-test comparing functional limitations (stair use) between participants with and without plantar fasciitis (n = 401).

Variable	Group	n	Mean	SD	t-value	df	*p*-value	Cohen’s d
Functional limitation (stairs)	Has PF	90	1.30	1.13	7.18	399	< 0.001	0.78
	Does not have PF	311	0.53	0.89				

Similarly, an independent-samples t-test comparing QoL scores showed that participants with PF reported significantly lower QOL scores (M = 6.26, SD = 3.02) than those without PF (M = 1.97, SD = 2.19), t(399) = -14.90, *p* < 0.001. The effect size was large (Cohen’s d = 1.63), indicating a strong difference between the groups (Table 7).

**Table 7:** Independent-samples t-test comparing quality of life scores between participants with and without plantar fasciitis (n = 401).

Variable	Group	Frequency (n)	Mean	SD	t-value	df	*p*-value	Cohen’s d
Quality of life	Has PF	90	6.26	3.02	− 14.903	399	< 0.001	1.78
	Does not have PF	311	1.97	2.19				

## Discussion

This study investigated the relationship between footwear choices and the prevalence of PF symptoms among adult women in Saudi Arabia, with a specific focus on identifying potential behavioral patterns and shoe-related factors contributing to PF. The prevalence of self-reported PF symptoms, especially pain after prolonged standing (58.4%), reflects a substantial burden, even though the most commonly worn shoe type was sports shoes (75.3%). Despite expectations that certain footwear types, particularly high heels or flat shoes, would strongly associate with PF symptoms, our findings did not reveal statistically significant associations between general shoe type and specific PF symptoms. This diverges from prior studies in the region that identified improper footwear as a clear contributing factor^5,12^. One explanation may be that while most participants wore sports shoes, not all athletic shoes offer sufficient arch support, cushioning, or fit consistency, and “sports shoe” as a category might mask internal variability in shoe quality.

Furthermore, although the frequency of high heel use was significantly associated with pain after prolonged standing (*p* = 0.047), the absence of broader significant associations across symptoms such as heel swelling, stabbing pain, and morning pain suggests that occasional or aesthetic-driven use of elevated footwear may contribute to cumulative loading stress without acting as a standalone etiological factor. These results are partially aligned with Barton et al., who found that high heels exacerbate forefoot pressure and gait asymmetry, though not all users develop PF^13^.

Our regression model indicated no significant predictive power of footwear-related variables on self-reported PF symptoms, contrasting with findings from Whittaker et al., where foot orthoses were effective in managing heel pain^14^. The near-significant odds ratio for use of cushioned insoles or orthotics in our study (*p* = 0.074) may reflect a reverse causality, in which individuals already experiencing PF symptoms seek orthotic relief. This highlights a key methodological insight: self-reported footwear and support device use may represent compensatory behaviors rather than true predictors of pathology onset.

Importantly, the absence of statistically significant associations between general shoe types and PF symptoms in this study does not negate the potential role of footwear. Rather, it highlights the multifactorial nature of PF, where a constellation of variables, including body weight, occupational standing time, anatomical predispositions, and physical activity levels, may mediate or confound the relationship. For example, elevated body weight increases plantar loading and strain on the fascia^15^, which could independently contribute to symptom development regardless of shoe type. In our study, although weight was reported (mean: 65.89 ± 17.23 kg), it was not adjusted for in the regression model, limiting our ability to disentangle its effect from that of footwear.

Furthermore, self-reported symptomatology captured by the FHSQ-Ar may reflect the cumulative burden of multiple lifestyle and biomechanical stressors rather than a single etiological factor. These limitations underscore the need for future studies to incorporate multivariate models controlling for body mass index (BMI), heel pad thickness, gait patterns, and duration of standing or walking per day. Such additions would allow for a clearer understanding of the independent and interactive effects of footwear on PF.

Notably, PF symptoms were associated with significant functional impairments. T-test results demonstrated that women with PF experienced greater limitations in stair usage and substantially lower quality of life scores, reinforcing the multidimensional impact of PF beyond localized foot pain. These findings corroborate those of Sullivan et al. and Alrashidi et al., who observed marked reductions in physical function and psychosocial well-being among individuals with plantar heel pain^6,16^.

Interestingly, although sandals are widely believed to be biomechanically inadequate, their underrepresentation in this study (1.7%) likely limited statistical power to detect associations. Similarly, only 2.7% reported high heels as primary footwear, underscoring a broader behavioral trend of preferring sports-style shoes. This raises a hypothesis: even within high-prevalence populations, shoe preference may already reflect pain-avoidant behaviors rather than risk-prone habits.

The discrepancy between the current findings and earlier literature may stem from differences in study design, outcome measurement, and population demographics. For instance, the self-reported nature of PF symptoms and footwear habits may introduce recall bias, particularly among those with intermittent or subclinical symptoms. However, given the relatively large sample (n = 401) and clear trends in pain and QoL data, the results remain informative and valid.

From a clinical perspective, the absence of strong footwear-PF associations suggests that clinicians and public health educators should broaden intervention strategies. Instead of focusing solely on footwear prescriptions, attention should be directed toward ergonomic education, load management, and occupational modifications, especially for populations exposed to prolonged standing. This view aligns with findings by Werner et al., who emphasized the role of workplace ergonomics and fatigue in musculoskeletal disorders^17^, and Belhan et al., who highlighted the influence of anatomical changes (e.g., heel fat pad thickness) on PF risk^5^.

Moreover, the culturally embedded use of both functional (sports shoes) and fashionable (heels, flats) footwear in Saudi Arabia requires context-specific education efforts. Future campaigns should target not only shoe choice but also duration of wear, surface exposure (e.g., tile, concrete), and the importance of alternating footwear to prevent load accumulation.

## Conclusion

PF is a prevalent and functionally limiting condition among adult women in Saudi Arabia, with over half of the participants reporting symptoms such as pain after prolonged standing. Although no statistically significant associations were found between general daily footwear type and self-reported PF symptoms, specific habits, such as frequent high heel use, were linked to increased discomfort following standing. Furthermore, participants with PF reported significantly lower quality of life and greater difficulty with physical functions such as stair use, highlighting the broader impact of the condition. These findings emphasize the multifactorial nature of PF and suggest that behavioral, anatomical, and occupational factors must be considered in prevention and management strategies. Culturally tailored education on footwear habits, ergonomic awareness, and early symptom recognition may help reduce the burden of PF in similar populations.

## Future work and limitations

There are various limitations on this study. First, because this study was cross-sectional, we were only able to evaluate relationships between shoe preferences and PF symptoms at one particular moment in time. As a result, it is impossible to prove a link between the intensity of heel pain and footwear characteristics.

Second, the diagnosis of PF was based on self-reported symptoms collected via a screening checklist, without clinical examination or imaging confirmation. While practical for large-scale online studies, this method introduces the possibility of misclassification.

Third, data were collected through a self-administered online questionnaire distributed via WhatsApp, which limited our ability to gather objective clinical data such as participants’ weight, height, or body mass index (BMI). These variables are known to be potential confounder factors.

Lastly, our exclusion criteria, while necessary to ensure participant safety and data relevance, may have limited the generalizability of our findings. For example, excluding individuals with comorbid conditions or foot surgeries.

Proposed strategies, future studies should adopted experimental designs to explore causal relationships between footwear choices and PF symptoms over time. To strengthen data accuracy future research should incorporate clinical assessments of foot alignment and biomechanical aspects. Future research directions should look at how well different kinds of footwear help to lessen plantar heel pain and enhance comfort during daily activities. To encourage proper footwear choices, it is also necessary to evaluate the general public’s awareness and attitudes on footwear and to create focused educational initiatives.

## Supplementary Information

Below is the link to the electronic supplementary material.

Supplementary Material 1

## Data Availability

The datasets generated and/or analyzed during the current study are not publicly available due to privacy restrictions, but are available from the corresponding author on reasonable request.
