# Supplementary material for: Footwear choices and their association with plantar fasciitis among adult women in Saudi Arabia: a cross-sectional study
Source: Sci Rep. 2025 Nov 17;15:40292. doi: 10.1038/s41598-025-24122-4 (PMC12623790; doi:10.1038/s41598-025-24122-4)
Supplement: Supplementary file 1 — Supplementary Material 1 [file 41598_2025_24122_MOESM1_ESM.pdf]

Footwear choices and their association with plantar fasciitis among adult women in Saudi Arabia: A cross-sectional study

Plantar fasciitis is a common foot condition resulting from lifestyle factors and repeated stress, particularly affecting middle-aged women who wear various types of footwear daily. This can lead to severe heel pain and, in advanced cases, plantar tears. Choosing appropriate footwear is essential for preventing inflammation and improving quality of life.
This study aims to evaluate the association between footwear choices and plantar fasciitis among middle-aged women, to determine its prevalence, explore the relationship between footwear characteristics and plantar fasciitis, and identify footwear-related features that may help reduce symptoms. The target age group is 20–40 years.

Participation in this research study is voluntary. Completing the questionnaire is expected to take no more than 10 minutes. Your name will not appear in the completed questionnaire, and no identifying information will be collected. You may withdraw at any time before completing the questionnaire. There are no anticipated risks associated with your participation. There are no costs or financial compensations involved. All information provided will remain anonymous, and all responses will be collected and analyzed collectively for research purposes only.

For inquiries, please contact the Investigator
Farah Alharbi – Email: 443000864@pnu.edu.sa

Section 1: Consent

Do you agree to participate in the research?

- Yes
- No

Section 2: Screening Questions

1. Is your age between 20 and 40?
   - Yes
   - No
2. Do you experience severe morning pain during your first steps after waking up?
   - Yes
   - No
3. Do you experience pain in the sole of your foot after standing for long periods?
   - Yes
   - No
4. Do you experience swelling or tenderness in the heel area?
   - Yes
   - No
5. Do you feel a sharp pain in the inner part of the lower heel?
   - Yes
   - No
6. Do you regularly wear shoes with thick soles or high heels?
   - Yes
   - No
7. Have you ever undergone foot surgery?

- Yes
- No

8. Do you suffer from muscular disorders (e.g., inflammation or pain)?
    - Yes
    - No
9. Do you have a history of plantar fasciitis?
    - Yes
    - No
10. Are you currently taking any prescribed medications for any of the following conditions: diabetes, osteoarthritis, hypertension, heart disease, lung disease, hormone replacement therapy, rheumatism, back pain, or depression?
    - Yes
    - No
11. Do you suffer from lumbar sciatica or neurological conditions affecting the foot?
    - Yes
    - No

Section 3: Personal Information

- Age: …………………
- Weight (kg): …………………
- Height (cm): …………………
- Occupation: …………………

Section 4: Type of Footwear

1. What type of shoes do you wear daily?
    - High heels (e.g., stiletto or medium heels)
    - Flat shoes (e.g., closed flat shoes)
    - Sports shoes
    - Sandals
    - Traditional shoes (e.g., house slippers)
    - Other (please specify): ……………………………
2. How many hours do you typically wear your main footwear daily?
    - Less than 2 hours
    - 2–4 hours
    - 5–7 hours
    - More than 7 hours
3. How often do you wear high heels?
    - Daily
    - Weekly
    - Occasionally
    - Never
4. What is the average heel height you usually wear?
    - Less than 2 cm
    - 2–5 cm
    - More than 5 cm
5. Do you use cushioned insoles or orthotics in your shoes?

- Yes
- No

Section 5: Foot Health Status

Note:
The following questions focus on your personal perception of your foot health.
Please choose the most appropriate answer. If unsure, select the option that is closest.

ng the Past Week

1. Pain level during the past week

○ None ○ Very mild ○ Mild ○ Moderate ○ Severe

Pain During the past week

1. Do you usually feel foot pain?

○ Never ○ Rarely ○ Sometimes ○ Often ○ Always

2.Do you usually feel persistent mild pain in your foot?

○ Never ○ Rarely ○ Sometimes ○ Often ○ Always

3.Do you usually feel sharp pain in your foot?

○ Never ○ Rarely ○ Sometimes ○ Often ○ Always

mpact of Foot Health on Daily Routine

During the past week

Has your foot caused you difficulties in performing work or activities?

Extremely ○ Quite a lot ○ Somewhat ○ A little ○ Not at all

Has your foot limited your performance of activities you usually do?

Extremely ○ Quite a lot ○ Somewhat ○ A little ○ Not at all

ring the past week:

To what extent has your foot health limited your walking?

Extremely ○ Quite a lot ○ Somewhat ○ A little ○ Not at all

To what extent has your foot health limited your ability to use stairs?

Extremely ○ Quite a lot ○ Somewhat ○ A little ○ Not at all

How would you rate your overall foot health?

Poor ○ Fair ○ Good ○ Very good ○ Excellent

xperience

ıg questions relate to your experience with footwear. Please select the option that best ur situation.

1.It is difficult to find shoes that do not hurt my feet.

○ Strongly agree ○ Agree ○ Neutral ○ Disagree ○ Strongly disagree

2.It is difficult to find shoes that fit my feet.

○ Strongly agree ○ Agree ○ Neutral ○ Disagree ○ Strongly disagree

3.I am limited in the number of shoes I can wear.

○ Strongly agree ○ Agree ○ Neutral ○ Disagree ○ Strongly disagree

4.Overall, how are your feet currently?

○ Excellent ○ Very good ○ Good ○ Fair ○ Poor

5.How would you rate your overall health?

○ Very good ○ Fair ○ Poor

ies

ɡ items refer to activities you may perform during a typical day.
what extent does your health limit you in the following:

Activities	Not at all	Yes, limits me a little	Yes, limits me a lot
a. Engaging in vigorous activities such as running, lifting heavy objects, or participating in strenuous sports			
b. Engaging in moderate activities such as moving a table, vacuuming, or gardening			
c. Carrying groceries from the supermarket			
d. Climbing several flights of stairs			
e. Climbing one flight of stairs			
f. Bending, kneeling, or prostrating			
g. Walking more than 1 kilometer			
h. Walking 100 meters			
i. Bathing or dressing yourself			

Social Activities

During the past four weeks, has your physical or mental health interfered with your usual social activities with family, friends, neighbors, or at other social events?

○ Extremely ○ Quite a lot ○ Somewhat ○ A little ○ Not at all

Feelings and Well-Being

The following questions refer to how you have felt and how things have been for you during the past four weeks. Please select **one** response for each question that is closest to your experience.

During the past four weeks, how often:

	Never	Rarely	Sometimes	Most of the time	All the time
a. Have you felt tired?					
b. Have you felt full of energy and vitality?					
c. Have you felt exhausted?					
d. Have you had a lot of energy?					

Health and Social Functioning

During the past four weeks, how much of the time has your physical health or emotional problems interfered with your social activities (such as visiting friends or relatives, etc.)?

○ Always ○ Most of the time ○ Sometimes ○ Some of the time ○ Never

Health Perception

How true or false are each of the following statements regarding your health?

**Statements**	**True or mostly true**	**I don't know**	**False or mostly false**
a. I seem to get sick more easily than others.			
b. My health is as good as that of anyone I know.			
c. I expect my health to get worse.			
d. My health is excellent.			
